# p53 controls genomic stability and temporal differentiation of human neural stem cells and affects neural organization in human brain organoids

**DOI:** 10.1038/s41419-019-2208-7

**Published:** 2020-01-23

**Authors:** Ana Marin Navarro, Robin Johan Pronk, Astrid Tjitske van der Geest, Ganna Oliynyk, Ann Nordgren, Marie Arsenian-Henriksson, Anna Falk, Margareta Wilhelm

**Affiliations:** 1 Department of Microbiology, Tumor and Cell Biology (MTC), Karolinska Institutet, Biomedicum, B7, SE-171 65 Stockholm, Sweden; 2 Department of Neuroscience, Karolinska Institutet, Biomedicum, D7, SE-171 65 Stockholm, Sweden; 3 Department of Molecular Medicine and Surgery, Karolinska Institutet, SE-171 76 Stockholm, Sweden; 4 Department of Clinical Genetics, Karolinska University Hospital, Stockholm, Sweden

**Keywords:** Differentiation, Developmental neurogenesis, Neural stem cells, Neural progenitors, Induced pluripotent stem cells

## Abstract

In this study, we take advantage of human induced pluripotent stem (iPS) cell-derived neural stem cells and brain organoids to study the role of p53 during human brain development. We knocked down (KD) p53 in human neuroepithelial stem (NES) cells derived from iPS cells. Upon p53KD, NES cells rapidly show centrosome amplification and genomic instability. Furthermore, a reduced proliferation rate, downregulation of genes involved in oxidative phosphorylation (OXPHOS), and an upregulation of glycolytic capacity was apparent upon loss of p53. In addition, p53KD neural stem cells display an increased pace of differentiating into neurons and exhibit a phenotype corresponding to more mature neurons compared to control neurons. Using brain organoids, we modeled more specifically cortical neurogenesis. Here we found that p53 loss resulted in brain organoids with disorganized stem cell layer and reduced cortical progenitor cells and neurons. Similar to NES cells, neural progenitors isolated from brain organoids also show a downregulation in several OXPHOS genes. Taken together, this demonstrates an important role for p53 in controlling genomic stability of neural stem cells and regulation of neuronal differentiation, as well as maintaining structural organization and proper metabolic gene profile of neural progenitors in human brain organoids.

## Introduction

TP53 is a tumor-suppressor gene that is mutated or inactivated in >50% of all human cancers^1^. Known as the guardian of the genome^2^, p53 is upregulated and activated by stress responses like DNA damage or oncogene activation^3^. Activation of p53 induces multiple cellular processes, such as cell cycle arrest, apoptosis, and differentiation^3^. While p53 mainly has been studied for tumor-suppressor functions, its role during human brain development is less understood. p53 is ubiquitously expressed throughout the whole mouse brain during early embryogenesis^4,5^, nevertheless, p53 knock out (KO) mice were found to be developmentally normal, albeit succumbing to early onset of tumor formation^6^. Further analyses demonstrated that between 8% and 16% of p53KO females developed exencephaly^7,8^, indicating a possible role for p53 in neural development. In addition, maintaining correct p53 protein levels in the central nervous system (CNS) is imperative to avoid inducing aberrant apoptosis or cell cycle arrest^9,10^. The role of p53 in CNS development has mostly been studied in murine models and little is known about the biological role of p53 in human brain development. With the discovery of cellular reprogramming, the field of stem cell biology has been revolutionized^11^. Induced pluripotent stem (iPS) cells derived from human somatic cells have the potential to virtually differentiate into any cell type of the body including neuroepithelial stem (NES) cells^12,13^. NES cells represent a developmentally immature stem cell state with the ability to differentiate into neurons and glia^13^. Interestingly, NES cells can also be captured directly from the developing human brain demonstrating their existence during development^14^. Furthermore, it is possible to use iPS cells for generating three-dimensional (3D) brain organoids that are capable of mimicking neurodevelopment^15,16^. Brain organoids replicate key gene expression and epigenomic pathways operating during human neurogenesis^17,18^ and contain progenitor cell populations capable of organizing and differentiating into mature neurons^19^.

Here we use a dual system approach to study p53’s role in human neurogenesis in vitro. By combining the cellular resolution of two-dimensional (2D) monolayer human neural stem cells, with the context of human neural stem cells in more in vivo like brain organoids, we demonstrate that p53 plays an important role in governing human neurogenesis.

## Material and methods

### Cell culture conditions of iPS cells

iPS cells were cultured in Essential 8^TM^ medium (ThermoFisher Scientific, A1517001) on plates coated with human recombinant Laminin-521 (LN-521, 1:20 in phosphate-buffered saline (PBS), Biolamina, LN521-03). Passaging was performed using TrypLE Express 1X (ThermoFisher Scientific, 12604013). Cells were seeded in media supplemented with 10 μM Y27632 Rho-kinase inhibitor (ROCKi, Millipore, SCM075). To induce DNA damage, cells were incubated with 0.5 μM Etoposide (Merck, E1383) for 3 h before harvest.

### NES cell derivation from iPS cells

Derivation of AF22 is described in ref. ^13^, and derivation of C9 NES cells from iPS cells was performed as described in ref. ^12^ with some modifications. Briefly, 3.8 × 10^4^ iPS cells/cm^2^ were seeded on LN-521 (Biolamina, LN521-03) coated 12-well plates in Essential 8^TM^ medium with 10 μM ROCKi. Neural induction was initiated by adding Neural Induction Media (NI media); Knock out serum replacement (KOSR) medium containing Dulbecco’s modified Eagle’s medium (DMEM)/F12 + GlutaMax (ThermoFisher Scientific, 31331-028), 20% KOSR (ThermoFisher Scientific, 10828028), 1× Non-essential Amino Acids (NEAA; ThermoFisher Scientific, 11140-076), 1:500 2-mercapthoethanol (ThermoFisher Scientific, 31350-010), penicillin–streptomycin (ThermoFisher Scientific 15140-122) with 10 μM of SB-431542 (StemCell Technologies, 72232), 500 ng/ml of Noggin (PeproTech, 120-10C), and 3.3 μM CHIR99021 (StemCell Technologies, 72052). Cells were fed on a daily basis with NI media. After 5 days of differentiation, 1 × 10^6^ cells were counted and seeded on a freshly LN-521-coated 12-well plates (LN-521, 1:20 in PBS). SB421542 was removed from NI medium formulation and increasing levels of N2B27 medium containing DMEM/F12 + GlutaMax, Neurobasal (ThermoFisher Scientific,21103-049), 0.2% 2-mercapthoethanol (ThermoFisher Scientific, 31350-010), 1 × N2 (ThermoFisher Scientific, 17504-044), and B27 (1:500) (ThermoFisher Scientific, 17502-048) was added and totally replaced at the end of the induction following 25%, 50%, 75%, and 100% proportions in comparison to KOSR medium for the next following 7 days. After 12 days of differentiation, the cells were passaged and introduced to NES cell culture conditions (see “NES cell culture”).

### NES cell culture

NES cells (AF22 and C9) were grown in high-density monolayers (4 × 10^4^ cells/cm^2^). Cells were single-cell passaged using TrypLE Express (ThermoFisher Scientific, 12604054) to 0.1 mg Poly-L-ornithine/1 μg/ml laminin (Merck, P3655-100MG and L2020, respectively) coated plates in NES culture medium (DMEM/F12+GlutaMax ThermoFisher Scientific, 31331-028) with 1× N2 supplement (ThermoFisher Scientific, 17504-044), 0.1% B27 supplement (ThermoFisher Scientific, 17502-048), 10 ng/ml fibroblast growth factor 2 (FGF2; ThermoFisher Scientific, AA10-155), 10 ng/ml human epidermal growth factor (EGF; PeproTech, AF100-15), and 1:100 penicillin–streptomycin (ThermoFisher Scientific, 15140-122) with puromycin (1 μg/ml) for transduced cells. To induce DNA damage, NES cells were incubated with 1 μM Etoposide (Merck, E1383) for 6 h before harvesting for protein lysate preparation.

### Production of lentivirus particles

Cells with stable knock down (KD) of p53 were generated by lentiviral transduction using short hairpin RNA (shRNA) against p53. shRNA p53#1 was constructed by cloning a hairpin oligo 5′-CCGGGACTCCAGTGGTAATCTACTTCAAGAGAGTAGATTACCACTGGAGTCTTTTTG-3′ and antisense shp53 AATTCAAAAAGACTCCAGTGGTAATCTACTCTCTTGAAGTAGATTACCACTGGAGTC into pLKO.1-puro vector. shRNA p53#2 and shRNA Control was obtained from Addgene (pLKO-p53-shRNA-941 was a gift from Todd Waldman, Addgene plasmid # 25637^20^) and #26701; pLKO.1-blast-SCRAMBLE was a gift from Keith Mostov (Addgene plasmid # 26701^21^). After sequencing and verification of correct insert, lentivirus was produced in HEK293T cells using packaging and envelope constructs pCMVΔ8.2 and pMD.G-VSV-G (pLKO.1-puro, pCMVΔ8.2 and pMD.G-VSV-G were gifts from Bob Weinberg (Addgene plasmids #8453, #8454, #8455)^22^. Briefly, HEK293T cells were seeded in 10 cm plates and transfected using calcium phosphate method; 20 µg of each pLKO.1 vector, 15 µg of packaging plasmid, and 6 µg envelope plasmid was used for transfection. Media was changed after 8 h. Twenty-four hours after transfection, viral supernatant was collected and concentrated using fast-trap virus purification and concentration kit according to the manufacturer’s instructions (Millipore). iPS and NES cells were transduced with lentiviral particles containing shRNA oligos diluted in iPS media or NES media, respectively. Transduced cells were selected with puromycin (1 μg/ml) starting 1 day after transduction. Cells were expanded when confluent.

### Proliferation assay

NES cells were seeded at 15,000 cells/cm^2^ in triplicates on 0.1 mg Poly-L-ornithine/1 μg/ml laminin-coated 24-well plates. Cell number was determined by cell counting each day for up to 4 days using the Bio-Rad TC20 counter.

### Neuronal differentiation

Non-guided neuronal differentiation was initiated by removal of growth factors EGF and FGF2 from the media and during the course of 2 weeks neurons are emerging. Media was changed every second day. Differentiation medium contained DMEM/F12+GlutaMax (ThermoFisher Scientific, 31331-028) with N2 supplement 1× (ThermoFisher, 17504-044), and 1% B27 supplement (ThermoFisher Scientific, 17502-048) with puromycin (1 μg/ml) for transduced cells.

### Quantitative real-time PCR

Total RNA was prepared using the Zymo Kit (Zymo, R1050). Total RNA (1 μg) was used for cDNA synthesis using Superscript III according to the manufacturer’s instructions (ThermoFisher Scientific, 18080-051). Real-time PCR using SYBR green dye was carried out on an ABI StepOnePlus (Applied Biosystems). Samples were run in triplicate and normalized to 28S or β2M. Relative expression was calculated using the ΔΔCT method. Primers are listed in Supplementary Table 1.

### Flow cytometry

To assess pluripotency, iPS cells were washed in 1 × PBS, dissociated with TrypleE Express, incubated with primary antibodies, and analyzed by FACS Celesta or LSR II (BD Biosciences). Antibodies used are shown in Supplementary Table 2. Proliferation rate of NES cells was analyzed by EdU (5-ethynyl-2’-deoxyuridine) incorporation and detected by the Click-iT EdU Flow Cytometry Assay Kit (ThermoFisher Scientific, C10635). The cells were grown in a coated (Poly-L-ornithine/Laminin) 6-well cell culture dish, pulsed with 10 µM EdU for 90 min, harvested, stained according to the manufacturer’s instructions, and analyzed by FACS calibur (BD Biosciences). Cell cycle analysis was performed by the FlowJo software. See Supplementary Table 2 for antibody information. For EdU cell cycle analysis of organoids, the same method and incubation time were used with the alteration of dissociating the organoid following the protocol described in the paragraph: “Isolation of PSA-NCAM+ neurons from whole-brain organoids.” To assess DNA damage-induced apoptosis, NES cells were treated with 1 μM Etoposide for 6 h before harvesting. Cells were stained with Annexin V/propidium iodide according to the manufacturer’s instructions (BD Biosciences, #556419), sorted by FACS calibur, and analyzed using FlowJo.

### Immunocytochemistry

Cells were grown on coated coverslips, washed with PBS, and fixed in 4% formaldehyde (VWR, 9713.1000) for 10 min. After washing multiple times, the coverslips were blocked in PBS containing 0.2% Triton-X100 (Sigma, T8787-50 ML) and 10% normal donkey serum (NDS; Sigma, D9663-10 ML) or normal horse serum (NHS; Sigma, H1138) at room temperature (RT) for 1 h. Next, coverslips were incubated with primary antibodies (see Supplementary Table 2 for a list of antibodies and dilutions) buffer containing 0.02% Triton-X100 and 1% NDS at 4 °C overnight. After washing multiple times with PBS, the coverslips were incubated with the appropriate secondary Alexa Fluor 488 or 594 (ThermoFisher Scientific) antibodies in PBS/0.02% Tween-20 with DAPI (4,6-diamidino-2-phenylindole; Sigma, D9542-10 mg) at RT for 1 h. After washing, the slides were mounted in fluorescent mounting solution (Dako, S302380-2).

### Western blot

Whole-cell lysates were prepared by lysing cell pellets in 1×NuPAGE LDS Sample buffer including protease and phosphatase inhibitor (ThermoFisher Scientific, 78440), incubated 5 min at 90 °C and 3 rounds of sonication for 5 min. Total protein was loaded on a 4–12% gradient Bis/Tris gel (ThermoFisher Scientific, NP0322BOX). Proteins were transferred to a nitrocellulose membrane, using the Trans-Blot Turbo Transfer System (Bio-Rad, 1704150). Antibodies and dilutions used are described in Supplementary Table 2.

### RNA preparation for Affymetrix Microarray

Total RNA was extracted using column-based Quick-RNA™ MiniPrep (Zymo, R1050), and RNA quality was assessed by using the Agilent Model 2100 Bioanalyzer (Agilent Technologies). 150 ng of total RNA was used to generate amplified sense strand DNA targets with Affymetrix WT Plus Reagent Kit followed by fragmentation and biotinylation with Affymetrix WT Plus Reagent Kit, and 2,5 μg of cRNA were hybridized for 16 h at 45 °C on Affymetrix HuGene-2.1st array. Gene expression data were analyzed using the Expression Console software (Affymetrix), only transcripts with entries in the Entrez Gene database were included in the sample group comparisons, and functional enrichment analysis was performed using gProfiler on a ranked list containing significantly changed genes (FC > 2, *p* < 0.05) (Supplementary Table 3) according to the Reimand protocol^23^, and the enriched pathways were visualized by Cytoscape EnrichmentMap and AutoAnnotate application^23,24^. Gene expression data have been deposited at Gene Expression Omnibus (GEO), accession number GSE141989.

### Cytogenetic analysis

NES cells (passages 30–35) were plated on poly-L-ornithine/laminin-coated 6-well plates and then treated with colcemid for 16–18 h. Following cell harvesting, metaphase slides were prepared according to standard procedures. Chromosome analysis was performed after GTG-banding with a resolution of approximately 550 bands per haploid genome.

### Extracellular flux metabolic analysis

Extracellular acidification rate (ECAR) and oxygen consumption rate (OCR) were measured using XFe96 Analyzer (Aligent Technologies) according to the manufacturer’s instructions. NES cells were plated at 60,000 or 80,000 cells/well density on 96-well plates (Seahorse cell culture plates) in NES or differentiation media (see culture conditions). One hour prior to assay was performed, medium was exchanged to XF assay medium pH 7.4, at 37 °C in a CO_2_-free incubator. Baseline levels were normalized to protein contents measured by DC protein assay (Biorad, 5000112) after the experiment was performed.

### Calcium imaging

For calcium imaging, differentiated cells were loaded with 5 mM Fluo-4 direct using Pluronic™ F-127 (both ThermoFisher Scientific F14201, P3000MP) and incubated for 20 min at 37 °C, after which the cells were washed 3 times using Krebs–Ringer’s solution, containing: NaCl (119 mM, S7653-250G), KCl (2.5 mM, P5405-250G), NaH_2_PO_4_ monobasic (1 mM, S3139-250G), CaCl_2_×2H_2_O (2.5 mM, C3306-100G), MgCl_2_×6H_2_O (1.3 mM, M2393-100G), HEPES (20 mM, H4034-100G), and d-Glucose (11 mM, G8270-100G; All from Sigma). This solution was adjusted to pH 7.4 at RT and pre-heated to 37 °C before washing the cells. Imaging was performed using a Zeiss Axio Examiner with the cells placed in a ring heater set to 37 °C. Frames were taken at 3 Hz for 20 min. Separate images were compiled into an image sequence and frames were aligned using Fiji. The image sequence was analyzed with FluoroSNNAP^25^. Regions of interest were manually drawn and kinetics of the calcium transients were calculated using Matlab (Matworks).

### Generation of whole-brain organoids from hiPS cells

Human iPS cells were maintained on LN521 in E8 media and passaged using TrypLE Express (ThermoFisher Scientific, 12604013). Cerebral organoids were generated as previously described^16,17,19^. Briefly, 9000 single iPS cells were plated in each round bottom well of a low-adhesive 96-well (Sigma, CLS7007-24EA). Every other day, half of the media was replaced until day 6, after which the media was changed to NI media (DMEM-F12:Neural Basal media 1:1, N2 supplement (1% vol/vol; all from ThermoFisher Scientific, 31331-028, 21103049, and 175020-01 respectively), GlutaMAX supplement (1% vol/vol; ThermoFisher Scientific, 35050-061), and MEM-NEAA (1% vol/vol; Sigma, M7145—100 ml), penicillin–streptomycin (1% vol/vol ThermoFisher Scientific, 15140-122), and heparin (1 μg/ml; Sigma, H3149-10KU). Medium was replaced every other day until clear pseudostratified epithelium emerged. When pseudostratified epithelium was visible, aggregates were transferred to growth factor reduced matrigel droplets (VWR, 734-0269) in 60-mm culture dishes as described in detail in ref. ^16^. After 4 days of static culture, a switch from NI medium to cerebral differentiation medium (DMEM-F12:Neural Basal medium 1:1, N2 supplement 0.5% (vol/vol), insulin 0.025% (vol/vol; Sigma, I9278-5ML), GlutaMAX supplement 1% (vol/vol), MEM-NEAA 0.5% (vol/vol) and penicillin–streptomycin 1% (vol/vol), 1:300,000 2-mercaptoethanol (0.16 μM; Gibco, 31350-010), and 2% (vol/vol; ThermoFisher Scientific, 12587010) B27 supplement without vitamin A was made. At day 20, media was changed to cerebral differentiation medium with 2% B27 supplement with vitamin A (ThermoFisher Scientific, 17504001) and placed on an orbital shaker, rotating at 60 rpm. At selected time points, organoids were washed with DPBS and fixed with 4% formaldehyde (VWR, 9713.1000). After fixation, organoids were placed in 30% sucrose (Sigma, S7903) and stored at 4 °C overnight, after which organoids were embedded using OCT (Sakura, 4530) and stored at −80 °C until sectioning.

### Isolation of PSA-NCAM+ neurons from whole-brain organoids

Dissociation of the organoids and isolation of immature neurons was performed using a neural dissociation kit (Miltenyi Biotec, 130-093-231) with some modifications. In short, after 30 days of differentiation, organoids were washed with cold PBS before incubation at 37 °C with trypsin for 5 min on an orbital shaker. After mechanical dissociation, addition of DNAse, and 5 extra minutes at 37 °C on the orbital shaker, cell viability was checked. Single-cell suspension was used to isolate the PSA-NCAM+ neurons according to the manufacturer’s instructions (Miltenyi Biotec, 130-092-966).

### Immunohistochemistry

For immunofluorescent staining, 20-μm serial sections were captured on superfrost+ glasses (Thermo Scientific, 10149870) using a cryostar NX70 (Thermo Scientific). Slides were dried 1 h at RT and stored at −20 °C before staining. Slides were washed in PBS/0.05% Tween-20 (Sigma) and incubated in PBS containing 10% NHS (Sigma, H1138) and 0.2% NP-40 (Sigma) at RT for 30 min to block non-specific binding, followed by incubation in a moist chamber with primary antibodies (for list of antibodies and dilutions used, see Supplementary Table 4) in PBS containing 1% NHS and 0.02% NP-40 at 4 °C overnight. After washing with PBS/0.05% Tween-20 (Sigma, P9416-100 ML), sections were incubated with the appropriate secondary Alexa Fluor 488, 594, or 647 (Invitrogen) antibodies in PBS/0.05% Tween-20 with DAPI (Sigma, D9542-10 mg) at RT for 1 h. After washing, the slides were mounted in fluorescent mounting solution (Dako, S302380-2).

### Image analysis

Pictures were taken with a Zeiss Axioskop 2 Plus epifluorescence microscope. Every image was captured with the same exposure time for p53KD and control samples. Images were analyzed in ImageJ. An automated counting method using the “Moments” threshold, which is provided in the software, was used to determine the total area covered by the cells by counting the number of pixels. To determine the number of TBR1+ and TBR2+ cells relative to the total number of cells, the total area of stained nuclei was determined for both antibodies and divided by the total area of all nuclei visualized by DAPI staining. To determine the number of KI67+ nuclei relative to SOX2+ nuclei or all nuclei of all cells, the total area of stained cells was measured for every image and then the KI67+ area was divided by the SOX2+ or DAPI+ area. Tiled imaged were made using a Zeiss LMS-800-airy confocal.

### Statistical methods

All results are represented as mean ± SD. For comparison between two groups, two-tailed Student’s *t* test was used. For comparing two or more groups, one-way analysis of variance with Dunnett’s post hoc was used. Sample size is stated in the figure legends. Statistical test assumptions were followed and values <0.05 were considered significant, with ***p < 0.0001; **0.001 < *p* < 0.01, and *0.01 < *p* < 0.05.

## Results

### Loss of p53 impairs neural stem cell division and promotes genomic instability

**Fig. 1:**
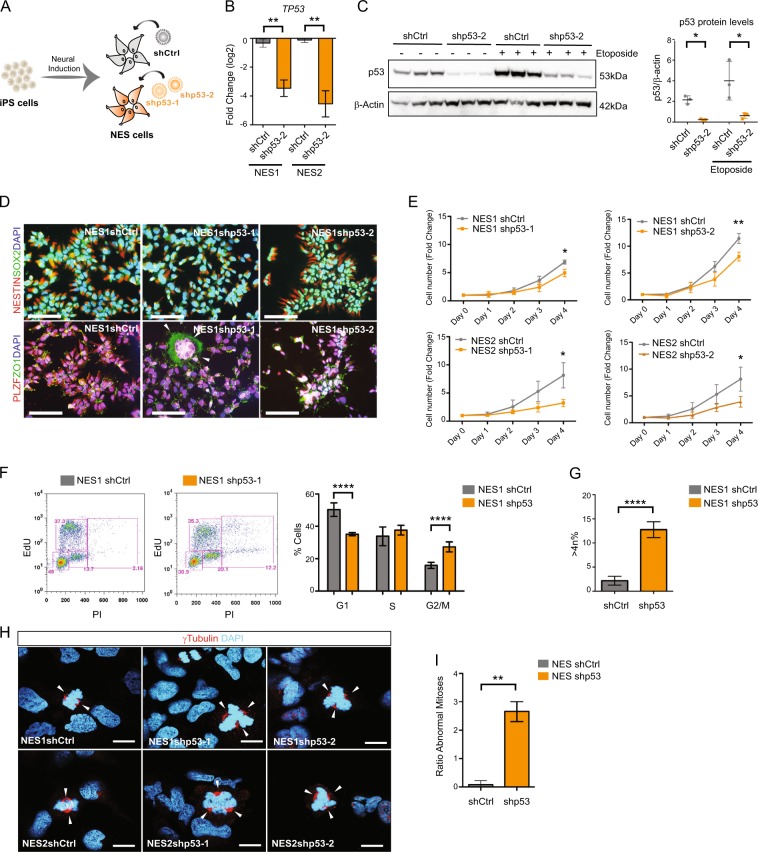
Loss of p53 impairs neural stem cell proliferation and promotes genomic instability. **a** Schematic outline of NES cell generation from iPS and shRNA transduction. **b** qRT-PCR validation of downregulation of *TP53* mRNA in NES1 shp53-2 and NES2 shp53-2. *n* = 3 independent experiments, **p < 0.01. Values are presented as mean ± SD. **c** Western blot and quantification of p53 protein levels in NES1 cells before and after 6 h 1 μM Etoposide treatment, *n* = 3 independent experiments. Values normalized to β-actin and presented as mean ± SD, *p < 0.05. **d** Validation of neural stem cell marker expression with immunofluorescence. Representative images of NESTIN, SOX2 and PLZF, ZO-1 expression is shown in NES1shCtrl and shp53-1 and -2. Scale bar 100 µm. **e** Proliferation assay in NES1 shCtrl, NES1 shp53-1, NES1 shp53-2, NES2 shCtrl, NES2 shp53-1, and NES2 shp53-2 cells, *n* = 3 independent experiments, values are presented as mean ± SD, **p < 0.01, *p < 0.05. **f** Left panel: Representative EdU/PI flow cytometry plots showing the distribution of cells in G1, S, and G2/M phase in NES1 shCtrl and shp53 cells pulsed with EdU for 90 min. Right panel: Quantification of EdU/PI Flow cytometry showing significant increase of cells in G2/M phase in p53KD NES cells, *n* = 4 independent experiments, values are presented as mean ± SD, ****p < 0.0001. **g** Quantification of >4*n* population identified by PI flow cytometry, *n* = 4 independent experiments. Values are presented as mean ± SD, ****p < 0.0001. **h** Gamma-Tubulin (γ-TUB) staining (in red) demonstrates centrosomal amplification in p53KD NES, scale bar 15 µm. **i** Quantification of mitoses displaying >2 centrosomes during division, *n* = 3 experiments. Data are represented as mean ± SD, **p < 0.01.

To address the role of p53 in human neural development, we knocked down p53 using shRNA in two different NES cell lines, NES1 (AF22) and NES2 (C9), that we have derived from iPS cells generated from two different healthy individuals^13,26^. NES cells were transduced with lentiviral p53 shRNA constructs targeting two different regions of p53 (shp53-1, shp53-2) or shCtrl (Fig. 1a, and Supplementary Fig. 1a). Downregulation of TP53 mRNA and protein was confirmed by quantitative reverse transcription polymerase chain reaction (qRT-PCR) and western blot (Fig. 1b, c, Supplementary Fig. 1b). Induction of DNA damage by Etoposide treatment resulted in an increase of both total p53 protein and Ser15 phosphorylated p53 protein in shCtrl NES cells but not in shp53 (p53KD) NES cells (Fig. 1c and Supplementary Fig. 1c). Furthermore, the apoptotic response to Etoposide was significantly impaired upon loss of p53 (Supplementary Fig. 1d). NES cells grow in a characteristic rosette-like organization and express neural stem cell markers NESTIN, SOX2, PLZF, and ZO-1^13,14,27,28^ that were still present after p53 KD (Fig. 1d, Supplementary Fig. 1e) showing that p53KD NES cells maintain markers of stemness specific for neural stem cells. Interestingly, shortly after transduction, a subset of p53KD NES cells display a different morphology compared to control cells, with distinctly larger nucleus (Fig. 1d, arrowheads, Supplementary Fig. 1f). To further study the effect of p53 loss, we performed a proliferation assay and found that p53KD NES cells had a reduced proliferation rate compared to control cells (Fig. 1e). We investigate the effect on cell cycle in more detail by pulsing cells with EdU, analyzed by flow cytometry, and found an increase of cells in G2/M phase and a significant accumulation of >4*n* cells in p53KD NES (Fig. 1f, g). It has previously been shown that loss of p53 leads to hyperamplification of centrosomes^29^, which are essential regulators of cell division and their deregulation is linked to neurodevelopmental disorders^30^. To understand the cause of the reduced proliferation rate and accumulation of >4*n* cells occurring after p53KD, we stained for centrosome marker γ-tubulin (Fig. 1h). We could indeed observe centrosome amplification in p53KD NES cells thus resulting in a significant increase of spindle malformations during mitosis (Fig. 1i). In support of this, karyotyping of p53KD NES cells showed accumulation of chromosomal aberrations over time, including aneuploidy and chromosomal translocations (Supplementary Fig. 1g). Taken together, this demonstrates that p53 is essential for maintaining proper cell division of human neural stem cells and deregulation affects proliferation, apoptotic response, and genomic stability of the stem cell pool.

### p53KD transcriptome analysis shows neural differentiation and metabolism as major affected pathways in human NES cells

**Fig. 2:**
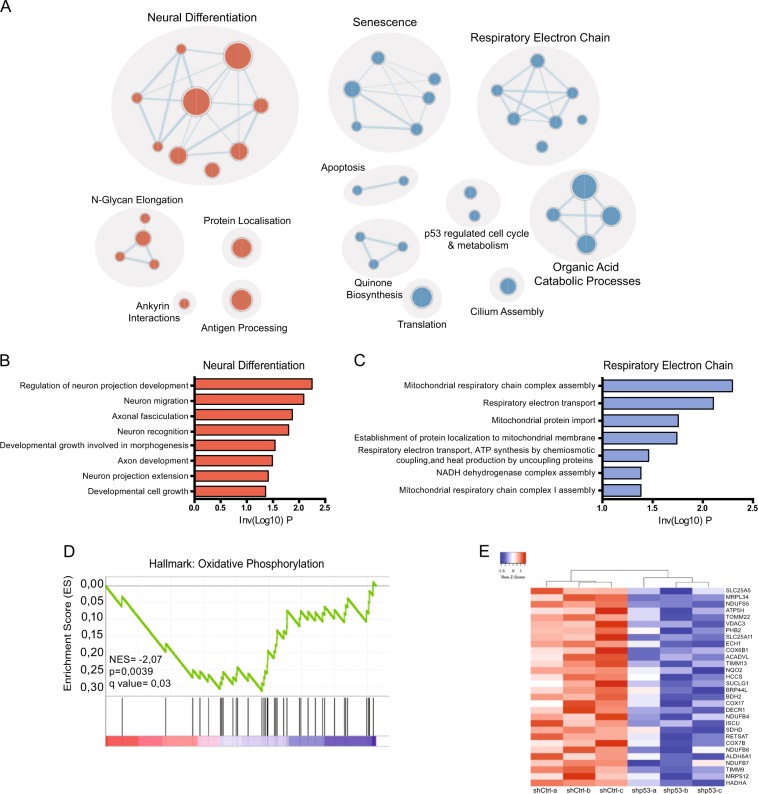
Neural differentiation and metabolic pathways are deregulated in p53KD NES cells. **a** Enrichment analysis of significantly changed genes (*p* < 0.05, FC ≥ 2) using gProfiler and cytoscape of biological pathways significantly deregulated in proliferating NES1 shp53-1 cells compared to shCtrl NES1 cells, *n* = 3 independent transductions. **b** Upregulation of biological pathways involved in neural differentiation. **c** Downregulation of biological pathways involved in respiratory electron chain and metabolism. **d** Gene set enrichment analysis showing enrichment plot of oxidative phosphorylation (OXPHOS) gene set. **e** Heatmap showing significantly downregulated oxidative phosphorylation genes in NES1 shp53-1 cells compared to control cells.

**Fig. 3:**
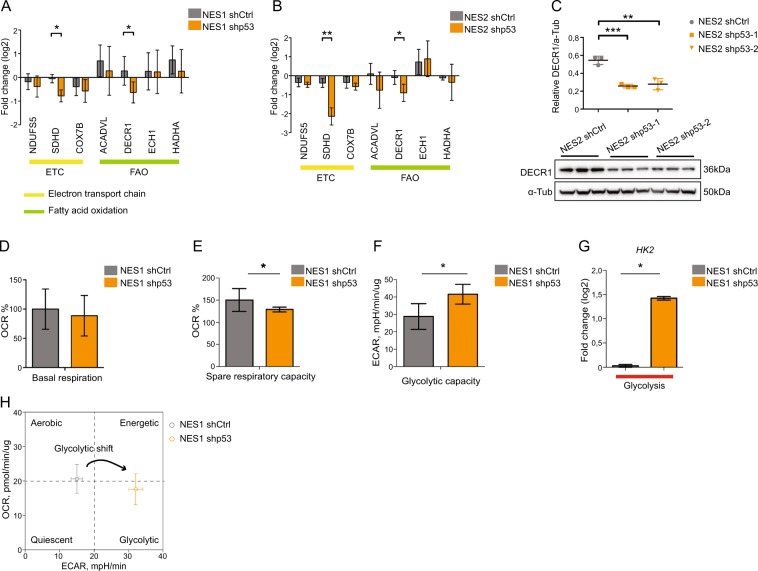
p53 maintains a metabolic balance in human neural stem cells. **a**, **b** Validation of a subset of OXPHOS genes in NES1 shp53 *n* = 7 (**a**) and NES2 shp53 *n* = 5 (**b**) cells using qRT-PCR, values are presented as mean ± SD, **p < 0.01, *p < 0.05. **c** Western blot analysis of DECR1 protein levels in NES shCtrl and shp53 cells, *n* = 3 independent experiments, and quantification of DECR1 protein normalized to α-Tubulin levels, values are presented as mean ± SD, ***p < 0.001, **p < 0.01. **d** Oxygen consumption rate (OCR) measurements for basal respiration and **e** spare respiratory capacity in p53KD NES cells, corresponding OCR level presented as percentage of baseline, *n* = 3 independent experiments, *p < 0.05. **f** Quantification of glycolytic capacity (ECAR) in p53KD NES cells, *n* = 3 independent experiments, *p < 0.05. **g** qRT-PCR analysis showing upregulation of *HK2* mRNA in p53KD NES cells compared to control cells, *n* = 3 independent experiments, *p < 0.05. **h** Quantification of basal respiration and cell energy phenotype chart representing the baseline metabolic status of NES control and p53KD NES cells visualizing the glycolytic shift. All data are presented as the mean ± SD.

To gain a greater understanding of which biological processes p53 controls in human neural stem cells, we analyzed the transcriptome of shp53-1 NES1 cells and found 2159 genes significantly deregulated (*p* < 0.05, FC ≥ 2) compared to shCtrl NES cells (Supplementary Table 3) including downregulation of p53 target, *CDKN1A*^31^ (*p21*; Supplementary Fig. 2a), while p53 family members *TP63* and *TP73* mRNA levels were not significantly changed (Supplementary Fig. 2b). Functional pathway enrichment analysis of significantly changed genes showed an upregulation of pathways involved in neuronal differentiation, while mitochondrial processes were downregulated (Fig. 2a–c, Supplementary Table 4). Using gene set enrichment analysis, we found genes involved in oxidative phosphorylation (OXPHOS) to be significantly reduced (Fig. 2d). In the OXPHOS cluster, several genes linked to fatty acid oxidation (FAO) and the electron transport chain (ETC) show significant downregulation (Fig. 2e). Both pathways are tightly linked to the tricarboxylic acid (TCA) cycle. FAO generates acetyl-CoA (A-CoA), which enters the TCA cycle, providing electron donors that are essential for ETC function. We could validate significant downregulation in mRNA levels of *SDHD* and *DECR1* in both NES1 and NES2 p53KD cells (Fig. 3a, b), as well as of DECR1 protein levels (Fig. 3c). *DECR1* has previously been identified as a putative p53 target gene^32^ and encodes 2,4 dienoyl-CoA reductase, an enzyme involved in reducing polyunsaturated fatty enoyl-CoA esters to A-CoA^33^. *SDHD* encodes succinate dehydrogenase complex subunit D, located in complex II of the ETC that connect the ETC to TCA through the conversion of succinate to fumarate^34^. The downregulation of enzymes involved in both FAO and ETC functions suggest a change in NES cell metabolism upon KD of p53. To functionally validate the role of p53 in human neural stem cell metabolism, we used the Seahorse XFe96 analyzer to measure two energy producing pathways in the cell, mitochondrial respiratory activity measured by OCR and glycolysis measured by lactate release, resulting in increasing ECAR (Supplementary Fig. 3a). We could not observe any significant difference in basal respiration rate between p53KD cells and Ctrl NES (Fig. 3d). However, when uncoupling ETC using FCCP, which measures the cells ability to respond to energetic demand, we observed a significant decrease in spare respiratory capacity in p53KD NES cells compared to control cells (Fig. 3e and Supplementary Fig. 3b). In line with a decrease in OCR, we found increased glycolytic capacity (Fig. 3f) and higher expression of *HK2*, encoding Hexokinase2 that catalyzes the first step in glycolysis (Fig. 3g), indicating enhanced glycolysis in p53KD NES cells. To visualize the energy profile of p53KD NES cells, we plotted the basal respiration and glycolysis parameters together in an energy phenotype chart (Fig. 3h), showing enhanced glycolysis-mediated energy production in the absence of p53. Our results suggest that p53 is important for maintaining the balance between mitochondrial respiration and glycolysis in human neural stem cells, a feature previously described in mouse liver and human colon cancer cells^35^.

### p53 regulates neuronal differentiation kinetics and maturity of neurons

**Fig. 4:**
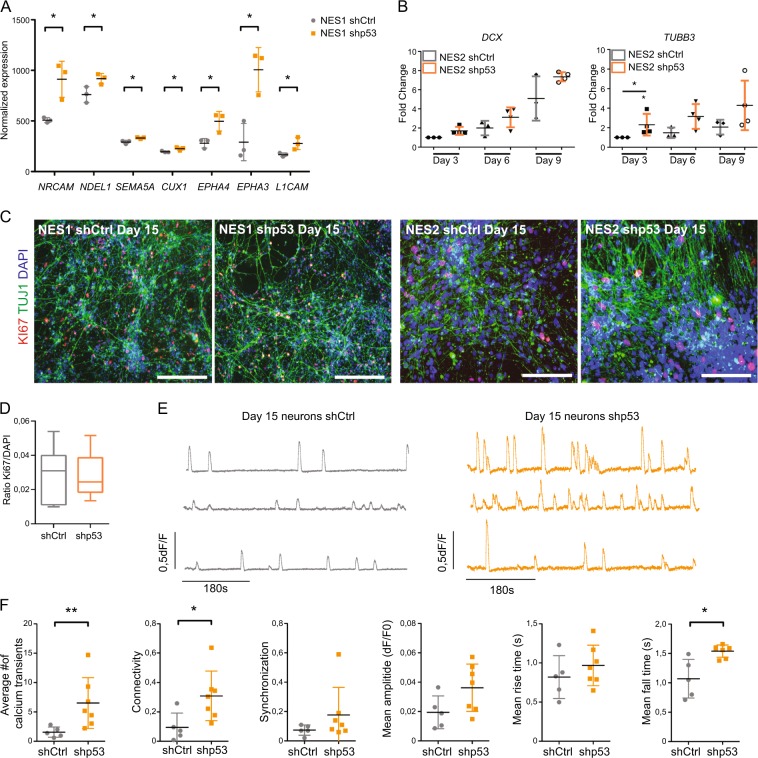
p53 regulates neuronal differentiation kinetics. **a** Gene expression from the transcriptomic analysis of NES1 shp53-1 cells compared to NES1 shCtrl cells for neuronal differentiation genes, values are presented as mean ± SD, *p < 0.05. **b** qRT-PCR analysis showing the expression of early neuronal genes *DCX* and *TUBB3* during differentiation days 3, 6, and 9. Pooled NES2 shp53-1 (*n* = 3) and NES2 shp53-2 (*n* = 1) was compared to NES2 shCtrl differentiated cells (*n* = 3), values are presented as mean ± SD, *p < 0.05. **c** Representative images showing TUJ1+ neurons (in green) after 15 days of differentiation intermingled with cycling cells (KI67+, in red), scale bar 100 μm. **d** Quantification of proliferating cells (KI67+/DAPI area) after 15 days of differentiation showing no differences between control neurons and p53KD neurons. Data represented as pooled shCtrl (*N* = 3) and shp53 (*N* = 3). **e**, **f** Calcium imaging analysis of 15-day-old neurons. **e** Representative traces of calcium oscillations. In gray, day 15 Ctrl neurons and, in orange, day 15 shp53 neurons. **f** Calcium kinetics with: average number of events per neuron over a 10-min period, mean fall time of a calcium transient in seconds, population-wide connectivity index, synchronization index, the average amplitude of the calcium transients and, mean rise time in seconds, of neurons having a simultaneous calcium transients. (See also movie S1 Ctrl neurons, and movie S2 p53KD neurons). Data represented as shCtrl is pooled NES1 shCtrl and NES2 shCtrl (*n* = 5) and shp53 is pooled NES1 shp53-1 and sh2 and NES2 shp53-1 and sh2 (n = 7), **p < 0.01, *p < 0.05.

Further analyses of the transcriptome data showed upregulation of several biological pathways involved in neural differentiation and we found upregulation of several pro-neuronal genes (Fig. 4a), suggesting that p53KD NES cells are primed toward a more differentiated state. To investigate whether this premature upregulation of neuronal differentiation genes would affect neurogenesis, we differentiated NES cells into neurons and analyzed the expression of early neuronal markers *TUBB3* and *DCX* at the indicated time points. We consistently observed an upregulation of both *DCX* and *TUBB3* in p53KD cells during the first 9 days of differentiation, however, we only observed a significant difference in *TUBB3* expression at day 3 (Fig. 4b), suggesting p53 as a regulator of early differentiation. Both p53KD NES cells and Ctrl NES cells were capable of generating Beta-III-TUBULIN (TUJ1; an epitope expressed on BETA-TUBULIN III) positive cells after 2 weeks of differentiation (Fig. 4c), showing that KD of p53 did not impede formation of neurons. As p53 is a cell cycle regulator, we quantified proliferating cells after 15 days of neuronal differentiation and found that there was no difference in the number of remaining proliferating cells (Fig. 4d), indicating that both p53KD and control cells were leaving the stem cell state in a similar manner. To further investigate whether the enhanced upregulation of neural differentiation genes observed in early neurons has a functional outcome on p53KD neurons, we used calcium imaging to assess the maturity of neuronal networks^36,37^. Representative calcium fluctuation traces are shown in Fig. 4e and (Supplementary video 1: shCtrl and Supplementary video 2: shp53). In line with an accelerated differentiation phenotype, p53KD neurons displayed more calcium transients and higher neuronal connectivity (Fig. 4f). Further analysis of the calcium transients showed neuronal network synchronization and increased amplitude and rise time of the calcium transients, although not significant, but that the fall time of calcium transients in p53KD was significantly longer (Fig. 4f), all together indicating a more mature calcium signaling profile. Next, we analyzed the expression of OXPHOS genes in the differentiated neurons, and although *TP53* is still stably downregulated during differentiation (Supplementary Fig. 4a), we could not observe any universal downregulation in OXPHOS gene expression (Supplementary Fig. 4b), suggesting that p53 is regulating metabolic balance in proliferating neural stem/progenitor cells but not in post-mitotic neurons.

### Loss of p53 results in unorganized stem cell structures in brain organoids and a reduction in TBR2+ intermediate progenitor cells and TBR1+ neurons

**Fig. 5:**
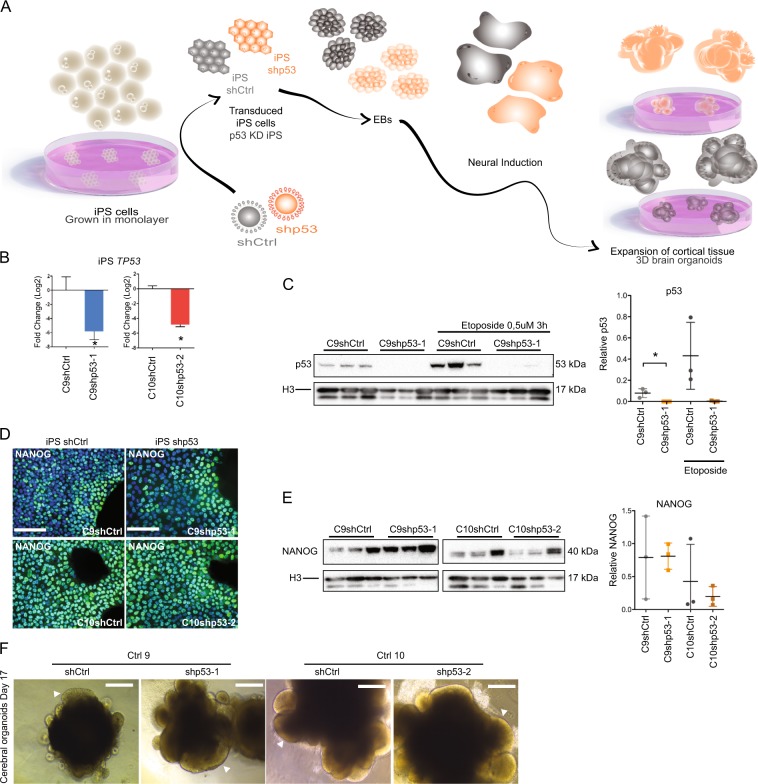
Generation of p53 knockdown brain organoids from iPS cells. **a** Illustration representing generation of whole-brain organoids from iPSCs transduced with short hairpin RNAs against p53. **b** qRT-PCR validation of p53KD of iPS cells. *n* = 3 independent experiments, values are presented as mean ± SD, *p < 0.05. **c** Western blot and quantification of p53 protein levels in iPS cells before and after 3 h 0.5 μM Etoposide treatment, *n* = 3 independent experiments. Values are normalized to H3 levels and presented as mean ± SD, *p < 0.05. **d** Representative images of validation of expression of pluripotency gene NANOG (green) in iPS shCtrl and iPS shp53 cells with DAPI (blue), scale bar 100 μm. **e** Western blot validation and quantification of NANOG protein levels in transduced iPS cell lines. Values are normalized to H3 levels and presented as mean ± SD. **f** Representative brightfield images of organoids on day 17 showing expanded neuroepithelium in all lines (arrowheads). Scale bar 100 μm.

To gain a better understanding of how the phenotypes we observed in p53KD NES would affect brain development in a more physiological system, we generated whole-brain organoids (Fig. 5a). Human iPS cells derived from two healthy individuals, CTRL-9 and CTRL-10 (from now on called C9 and C10, respectively)^38^, were transduced with shCtrl or shp53-1 or shp53-2, respectively, generating two p53KD lines (C9shp53-1 and C10shp53-2) and corresponding control lines, C9shCtrl and C10shCtrl (Supplementary Fig. 5a). We validated p53KD by both qRT-PCR and western blot (Fig. 5b, c Supplementary Fig. 5b). Furthermore, upon treatment with Etoposide, p53KD iPS cells showed impaired accumulation of total and phosphorylated p53 protein (Supplementary Fig. 5c). KD cells were assessed for pluripotency markers (OCT4, SSEA4, and TRA 1-60) by immunofluorescence or flow cytometry (Supplementary Fig. 5d, e). NANOG was determined by immunofluorescence and western blot analysis (Fig. 5d, e, Supplementary Fig. 5f), showing that the expression of key pluripotency markers was not affected by p53KD. We generated brain organoids from all four transduced iPS lines. Brightfield pictures show the generation of pseudostratified epithelium indicating that neuroepithelium was forming in controls and p53KD organoids after 17 days (Fig. 5f), demonstrating that p53KD iPS cells could differentiate into similar structures as control iPS cells.

**Fig. 6:**
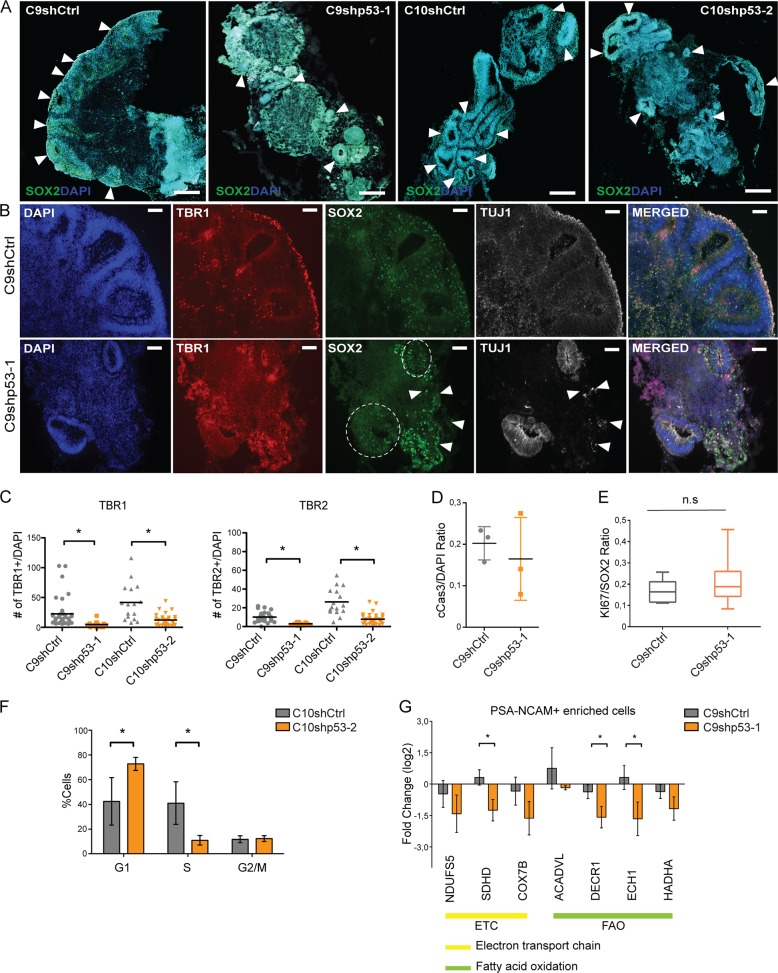
Unorganized stem cell structures and diminished neurogenesis in p53KD brain organoids coupled with downregulated OXPHOS genes. **a** Tiled image of representative control and p53KD organoids at day 30 of differentiation (D30). In control organoids, organized neural tubular regions are visible, whereas in p53KD organoids, generation of neural tissue was less efficient and overall organization severely disrupted. SOX2 is shown in green and nuclei were stained with DAPI (blue), scale bar 500 μm. **b** Representative images of C9shCtrl and C9shp53-1 D30 organoids. In shCtrl organoids, SOX2+ neural tubular regions are visible surrounded by TBR1+ TUJ1+ neurons. In contrast, C9shp53-1 organoids have SOX2+ cells scattered inside and outside the tubular regions (dotted circle), scale bar 100 μm. **c** Quantification of total TBR1+ and TBR2+ cells, normalized to DAPI, data are represented as mean ± SD, *p < 0.05. **d** Quantification of cleaved caspase 3 (cCas3) positive cells. **e** Quantification of SOX2+/KI67+ cells in neurogenic niches. **f** EdU cell cycle analysis of day 30 organoids pulsed with EdU for 90 min, *n* = 3 independent experiments. **g** qRT-PCR analysis of OXPHOS gene expression in C9 PSA-NCAM+ isolated neuronal progenitors from D30 organoids. Data are represented as mean ± SD, *p < 0.05.

After 30 days of differentiation (D30), we analyzed the organoids and detected reduced neural areas and a general disorganized appearance in p53KD organoids (Fig. 6a arrowheads). Furthermore, in control organoids there is an abundance of first cortical layer of TBR1+ post mitotic neurons, while their presence is significantly reduced in p53KD organoids (Fig. 6b, c, Supplementary Fig. 6a, c). To investigate whether the reduced number of neurons was due to a reduction of stem cells and progenitor cells, we analyzed the stem cell marker SOX2 and intermediate progenitor cell marker TBR2. Interestingly, we found that the neural stem cell layer is disorganized in p53KD organoids, with SOX2+ neural stem cells located both inside and outside tubular regions (Fig. 6b and Supplementary Fig. 6a). In relation to the disorganization of SOX2+ and TBR1+ cells, TUJ1 expression is observed throughout various regions in the organoid, always in proximity to SOX2+ cells but not necessarily in proximity to tubular regions (Fig. 6b). In addition, we observed a significant reduction of TBR2+ intermediate progenitors in the p53KD organoids (Fig. 6c, and Supplementary Fig. 6b). Next, we analyzed whether the reduction of TBR2+ intermediate progenitors was due to enhanced apoptosis or reduced proliferation of the SOX2+ stem cell population. We did not observe any significant changes in cleaved caspase 3+ cells (Fig. 6d, Supplementary Fig. 6d), suggesting no altered apoptosis in the p53KD brain organoids at this timepoint. In addition, we could not find any significant changes in KI67/SOX2 double positive cells (Fig. 6e, Supplementary Fig. 6e). However, KI67 is a broad cell cycle marker, labeling proliferating cells but not quiescent G_0_ cells. To investigate the cell cycle distribution in more detail, we pulsed D30 organoids with EdU, dissociated the organoids, and performed flow cytometric analysis. Interestingly, we observed an accumulation of cells in G1 phase and a reduction of cells in S phase (Fig. 6f, Supplementary Fig. 6f), suggesting reduced proliferation rate in the p53KD organoids. To evaluate the effect on metabolic genes observed in the NES cells, we isolated the PSA-NCAM+ neuronal progenitor population of the organoids and detected downregulation of several OXPHOS-related genes (Fig. 6g and Supplementary Fig. 6g, h). This indicates a role for p53 in regulating the metabolic profile of human brain organoid progenitors as well. Taken together, our data show that p53 has an important function in regulating proper human neurogenesis.

## Discussion

Here we report that p53 is required for proper human neurodevelopment. Our findings show that loss of p53 in human NES cells leads to centrosome amplification resulting in defective mitosis and chromosomal instability, confirming p53 as a key regulator of genomic integrity. p53 has previously been described to regulate stem cell proliferation, and loss of p53 results in enhanced proliferation of mouse neural stem cells in neurosphere assays^39–41^. Interestingly, in contrast to findings in mouse neural stem cells, we show that loss of p53 in otherwise healthy human neural stem cells resulted in slower proliferation. This was accompanied by an increase in aberrant mitoses with more than two centrosomes and an accumulation of cells in G2/M phase. Centrosomes organize the spindle formation during cell division^42^ and amplification of centrosomes can lead to a prolonged G2 phase^43^, suggesting that the reduced proliferation rate may be due to an inability of p53KD NES cells to proceed normally through the G2/M phase. In contrast, p53KD in the 3D organoid system, results in an accumulation of cells in G1 phase at the expense of S phase. Both systems indicate a reduced proliferation rate, affecting, however, a different part of the cell cycle. Differences in cell behavior between 2D and 3D cells culture systems have only recently been started to be explored. We have previously shown that MYC is mitogenic in chick radial glial precursors grown in 2D, while it drives neural differentiation when expressed in an intact neural tube^44^. Furthermore, it has been shown that AKT/mammalian target of rapamycin signaling is reduced when a human colon cancer cell line is transferred from 2D to 3D^45^, demonstrating the inherent differences between a 2D and 3D system.

The discrepancy between our results and previous reports regarding proliferation could be due to species differences. Recent studies have shown differences in mouse and human transcription factor regulation^46^. Only 44% of transcription factor regulatory circuitry between mouse and human overlap, and >1000 genes in the p53 regulatory network are differentially regulated between mouse and human^46,47^. Furthermore, NES cells resemble a human embryonic neural stem cell found around gestation weeks 5–7^14^, whereas previous studies have been performed on mouse postnatal radial glia-like stem cells^41^. Moreover, when p53KO E13 mouse brains were studied, no differences in proliferation markers compared to wild type were found^45^. In addition, a recent study shows that loss of UTX and 53BP1 severely affected human cortical development but not rodent neurogenesis^48^, indicating not only the relevance of the studied time point in regard to proliferation but also that there are species-specific regulatory networks governing neurogenesis. This highlights the importance of studying biological processes in relevant human cell models.

The transcriptome analysis of p53KD NES cells revealed OXPHOS among the significantly downregulated pathways. Metabolic pathways are tightly regulated in neural stem cells and govern important functions dictating differentiation cues and fate choices^49^. In healthy and in cancer cells, p53 is an important regulator of both glycolytic and oxidative metabolism, controlling energy demanding processes such as proliferation, growth, and survival^50,51^. We could validate downregulation of *SDHD* and *DECR1*, genes important for the ETC and FAO. The functional change of this dysregulation was assessed by measuring mitochondrial respiration and glycolysis levels, which revealed the increased use of glucose as an energy source of p53KD NES, showing p53 as regulator of metabolism in human neural stem cells. p53 has been extensively studied in its role in cancer metabolism^50^ and has been implicated in balancing mitochondrial respiration and the glycolytic pathways^35,52^. Next to an aberrant metabolism, we observed an upregulation of neuronal differentiation genes in p53KD NES cells, suggesting that p53 loss primes neural stem cells for differentiation, although not enough to leave the stem cell state, as shown by the persistence of neural stem cell markers NESTIN, SOX2, PLZF, and ZO-1. However, after removal of growth factors, accelerated neurogenesis of p53KD NES cells is apparent with subsequent upregulation of early neuronal differentiation genes leading to neurons with a more mature network formation, indicated by increased calcium transients. Interestingly, accelerated neurogenesis was previously shown in vivo after radiation of the dentate gyrus of p53KO mice^53^, as well as in neurospheres isolated from E13 p53KO mice^45^ and, differentiation of p53KO mesenchymal stem cells^54^. Translating the phenotypes of the NES cells to a more physiological brain organoid, we analyzed more specifically cortical neurogenesis. Here we observed a redistribution of SOX2+ neural stem cells in p53KD brain organoids, spread out in the organoid and not restricted to the neural tubular regions like in control organoids. Previous work has shown the loss of p53 together with the centrosomal protein CEP63 results in misplacement of SOX2+ cells in the mouse sub-ventricular zone^55^. This suggests a role for p53 and interacting partners in controlling correct neural progenitor arrangement. Furthermore, diminished neurogenesis was observed with a decrease in TBR2+ progenitor cells and TBR1+ neurons. Further analysis of OXPHOS genes in PSA-NCAM+ cells isolated from the organoids showed a downregulation of several genes involved in mitochondrial complex function. Mitochondrial complex function is required for intermediate progenitors like TBR2+ progenitors^56,57^. Interestingly, genetic inhibition of mitochondrial function has been shown to inhibit neurogenesis^58^. Therefore, with the downregulation of several genes in the ETC and FAO as shown in the NES cells and the PSA-NCAM+ progenitors derived from the organoids, we confirm the p53-mediated metabolic gene balance in human neural progenitors and speculate that decreased OXPHOS function could lead to the reduction of TBR2+ intermediate progenitors, an important regulator of neocortical formation^59,60^, and consequently a reduction of TBR1+ post-mitotic neurons.

Here we show the involvement of p53 in human neurogenesis. The loss of p53 in human neural stem cells contributes to genomic instability and metabolic changes, possibly due to the amplification of centrosomes and consequently impaired cell division. These aberrations in NES cells resulted in an accelerated differentiation with higher neuronal activity and a more mature phenotype for p53KD neurons. Translating aberrant neural stem cell function to in vitro cortical neurogenesis, we show that p53 aids in proper neural stem cell positioning and regulates neurogenesis, possibly due to mitochondrial function. Taken together, our 2D and 3D models derived from human iPS cells demonstrates the relevance of a multi-systems approach to elucidate the role of p53 in human neuronal development.

## Supplementary information

Supplemental Figure S1

Supplemental Figure S2

Supplemental Figure S3

Supplemental Figure S4

Supplemental Figure S5

Supplemental Figure S6

Movie 1

Movie 2

Supplemental Figure and Table legends

Supplemental Table 1

Supplemental Table 2

Supplemental Table 3

Supplemental Table 4
