# Supplementary figures and images for: p53 controls genomic stability and temporal differentiation of human neural stem cells and affects neural organization in human brain organoids
Source: Cell Death Dis. 2020 Jan 23;11(1):52. doi: 10.1038/s41419-019-2208-7 (PMC6978389; doi:10.1038/s41419-019-2208-7)

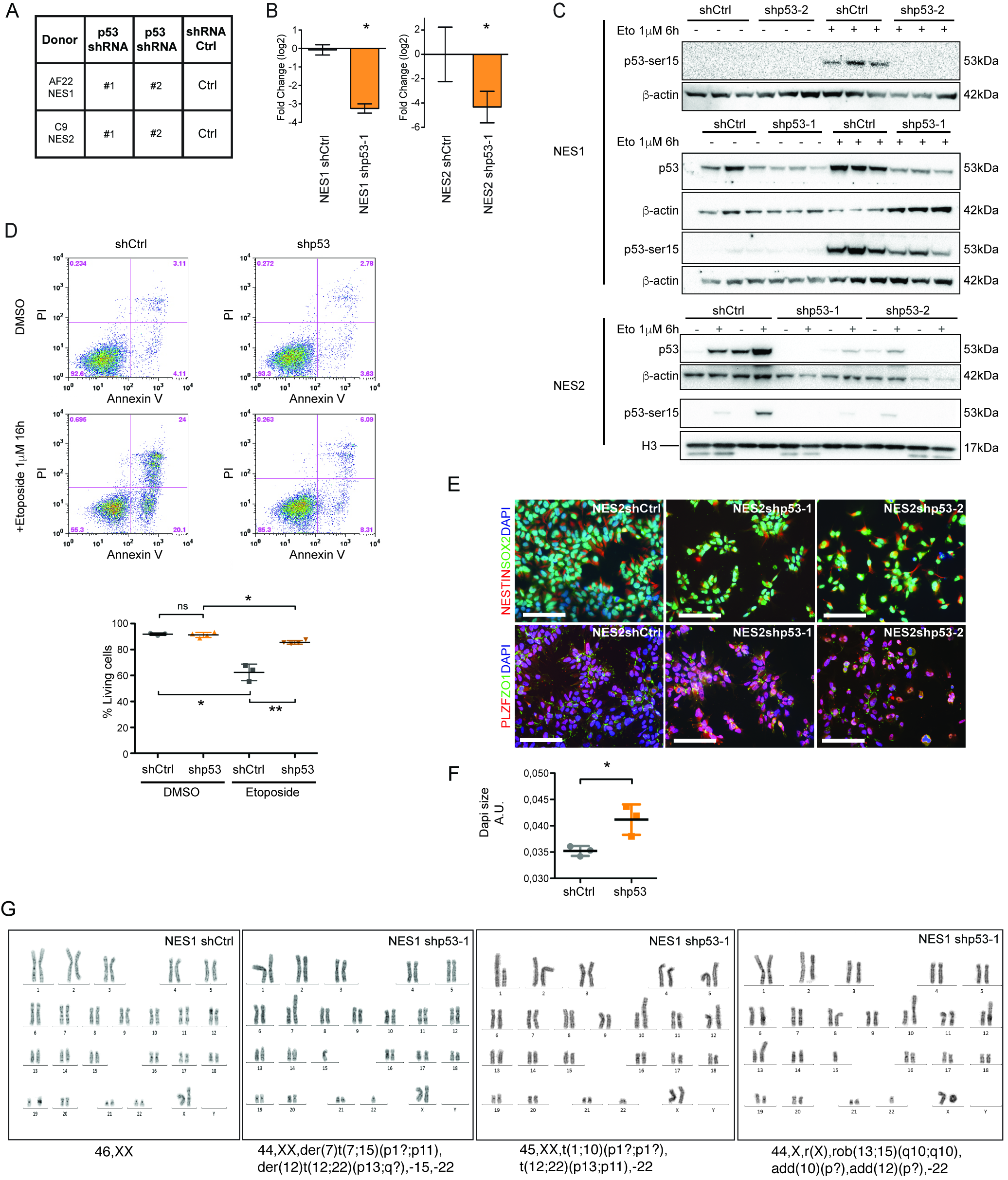

Supplement: Supplementary file 1 — Supplemental Figure S1 [file 41419_2019_2208_MOESM1_ESM.tif]

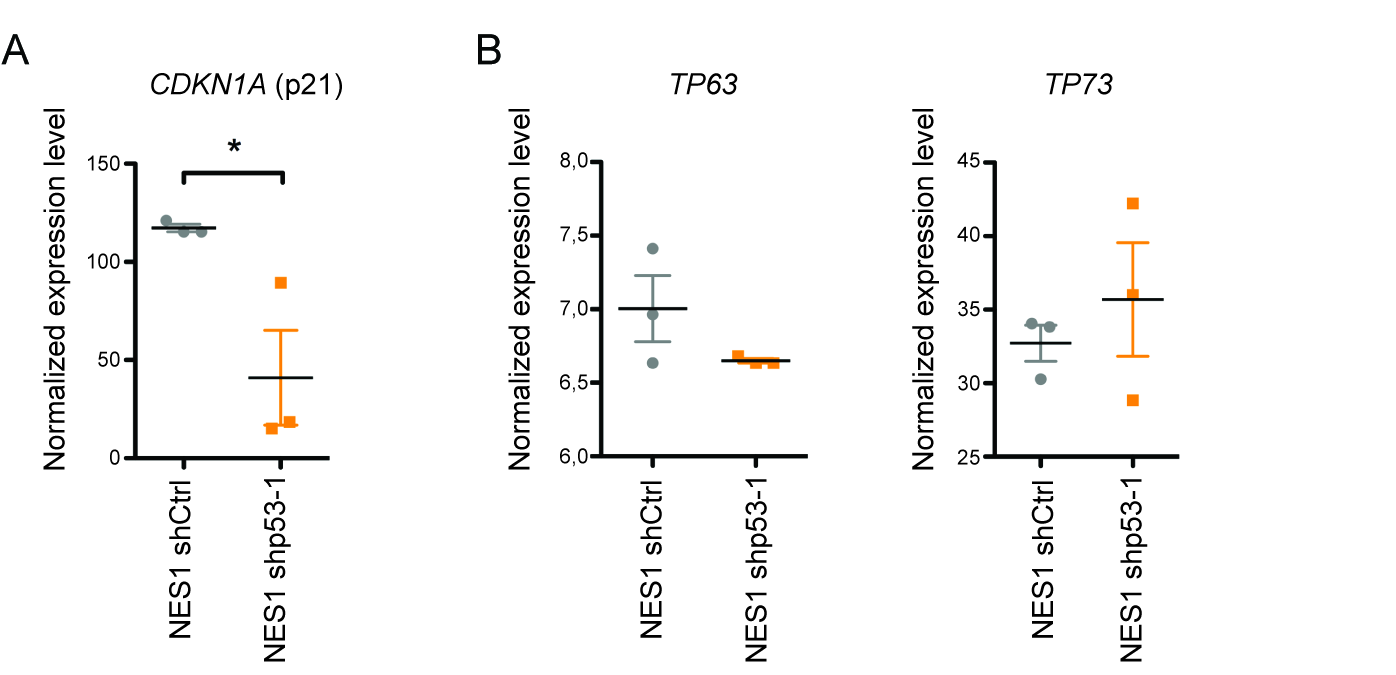

Supplement: Supplementary file 2 — Supplemental Figure S2 [file 41419_2019_2208_MOESM2_ESM.tif]

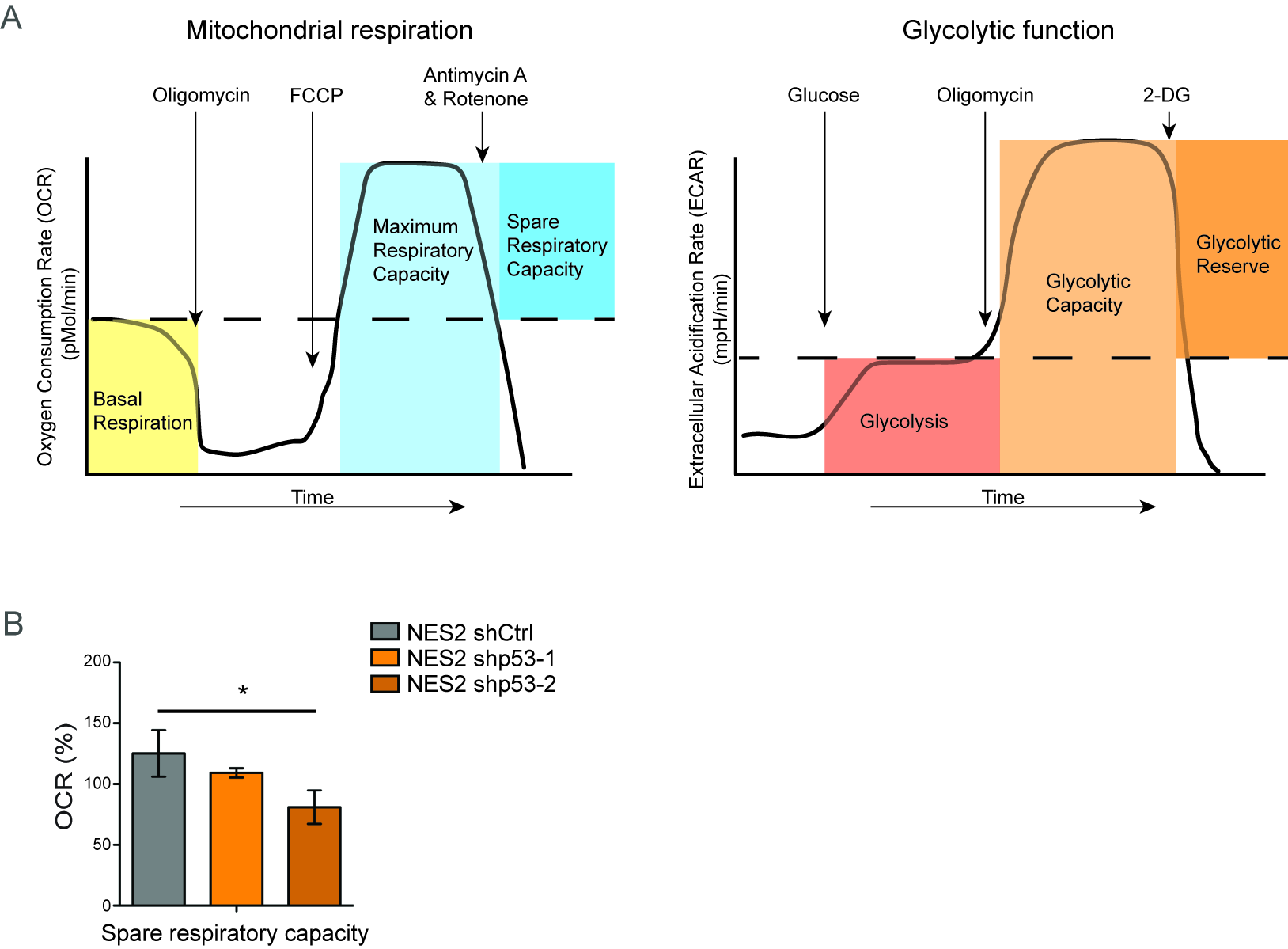

Supplement: Supplementary file 3 — Supplemental Figure S3 [file 41419_2019_2208_MOESM3_ESM.tif]

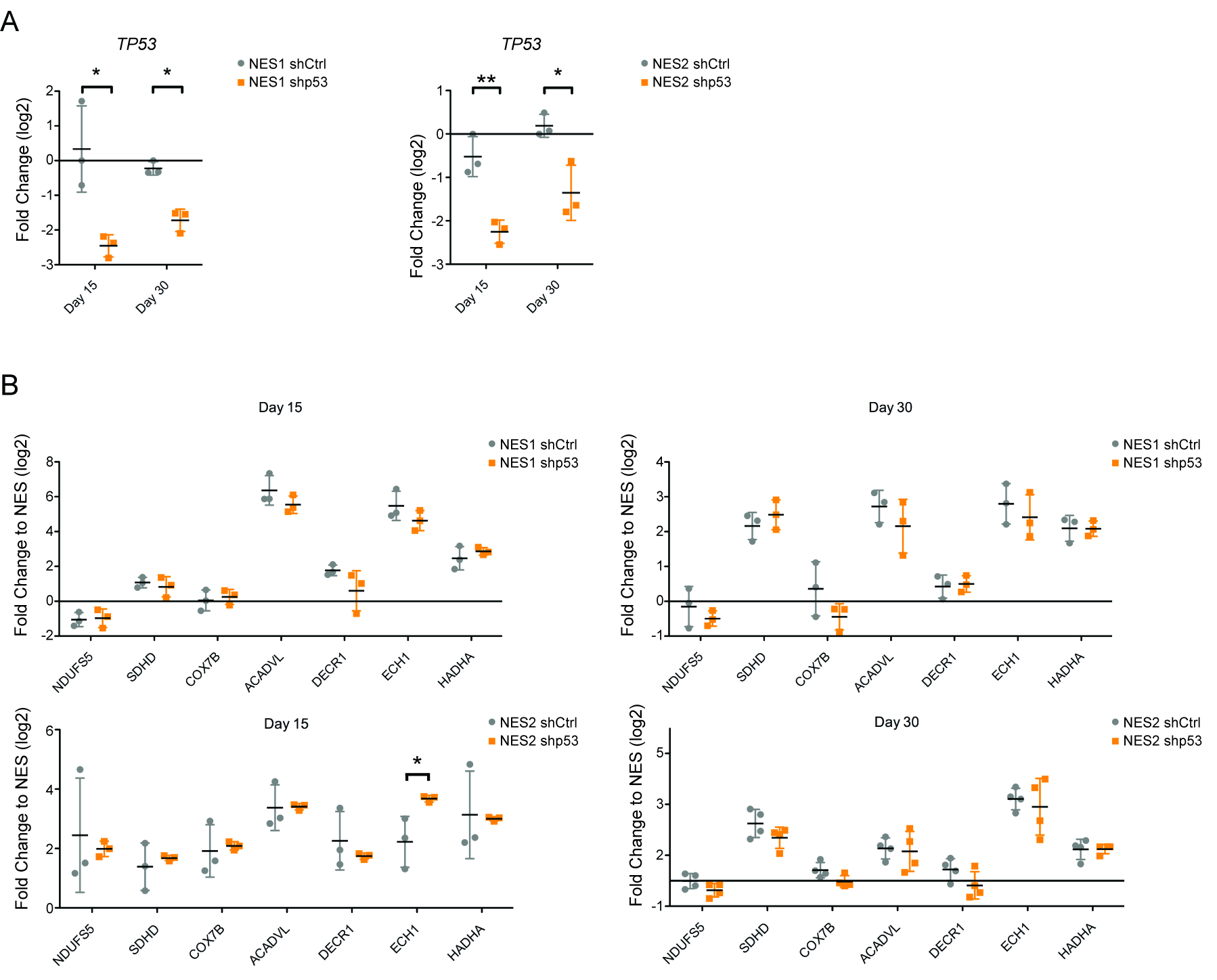

Supplement: Supplementary file 4 — Supplemental Figure S4 [file 41419_2019_2208_MOESM4_ESM.tif]

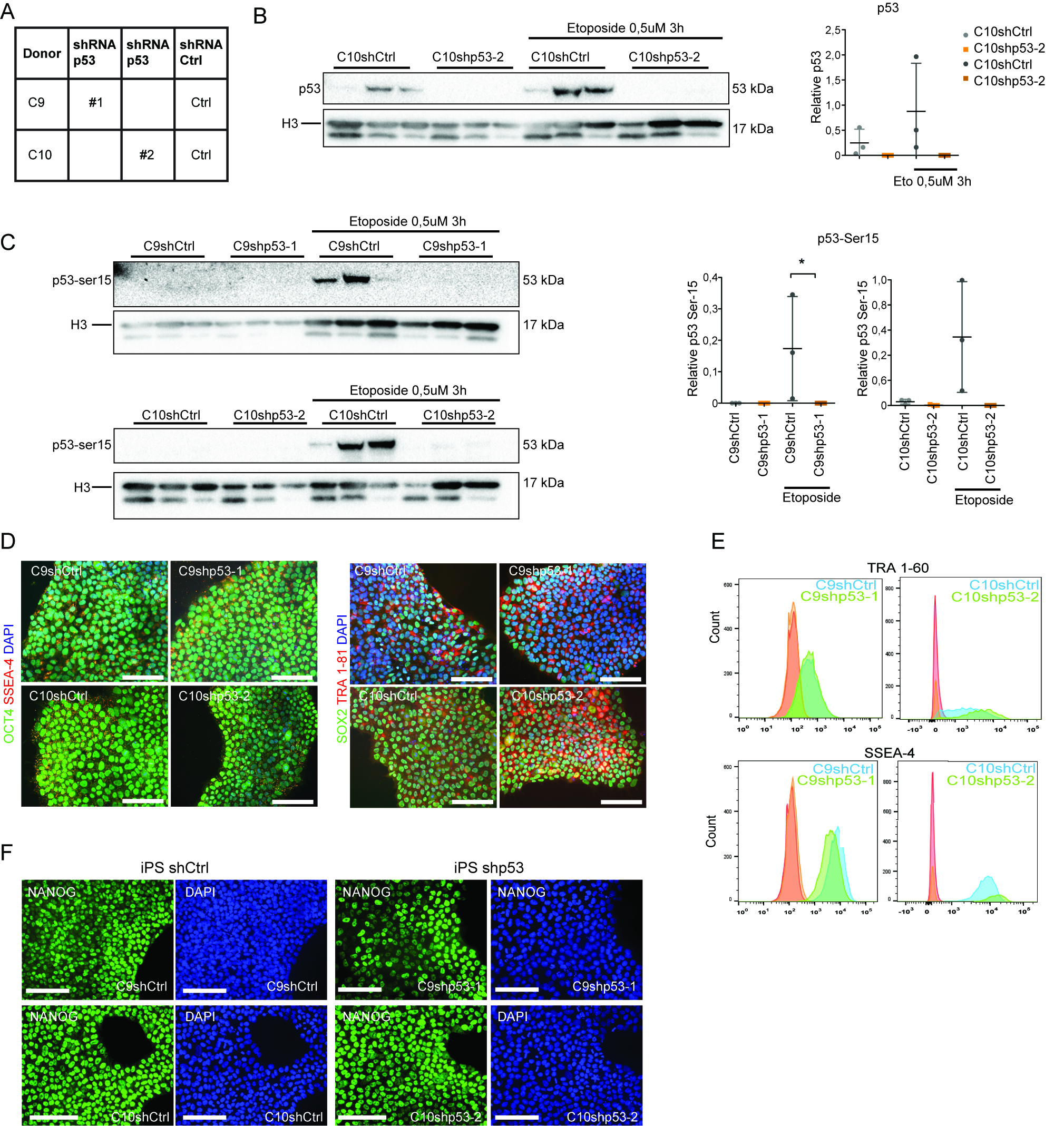

Supplement: Supplementary file 5 — Supplemental Figure S5 [file 41419_2019_2208_MOESM5_ESM.tif]

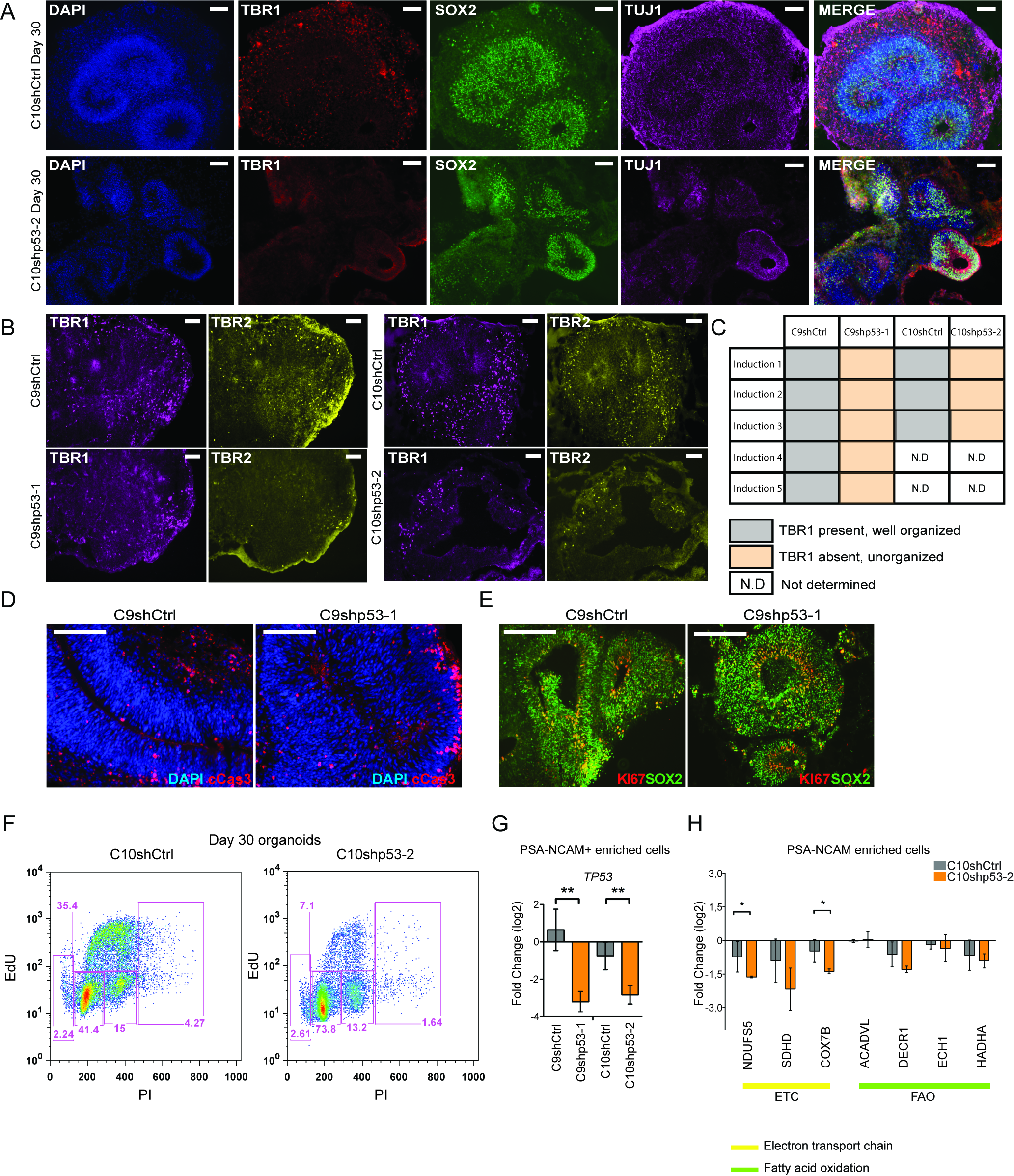

Supplement: Supplementary file 6 — Supplemental Figure S6 [file 41419_2019_2208_MOESM6_ESM.tif]
