# Supplementary material for: p53 controls genomic stability and temporal differentiation of human neural stem cells and affects neural organization in human brain organoids
Source: Cell Death Dis. 2020 Jan 23;11(1):52. doi: 10.1038/s41419-019-2208-7 (PMC6978389; doi:10.1038/s41419-019-2208-7)
Supplement: Supplementary file 9 — Supplemental Figure and Table legends [file 41419_2019_2208_MOESM9_ESM.docx]

**Supplemental Figure Legends**

**Supplemental Figure 1 (Related to Figure 1)** (A) Table of used NES cell lines and shRNA constructs. (B) qRT-PCR validation of p53KD in NES1 shp53-1 and NES2 shp53-1, *p<0,05 (C) Western blot analysis of NES1 and NES2 before and after Etoposide treatment (1μM for 6hours), n=3 experiments (D) Annexin V/PI flow cytometry analysis after 16 hour 1μM Etoposide treatment, axes represent % of living cells. shCtrl n=3, shp53 n=4. (E) Validation of neural stem cell identity with immunofluorescence. Expression of NESTIN, SOX2 and PLZF, ZO-1 is shown in NES2shCtrl and shp53-1 and -2. Scale bar 100 µm (F) Quantification of cell nucleus size of non-dividing cells n=3, *p<0,05. (G) Karyotype of NES1 shCtrl and NES1 shp53-1 with description of aberrations.

**Supplemental Figure 2 (Related to Figure 2)**

Data extracted from the transcriptomic analysis (A) showing downregulation of *CDKN1A* (p21). (B) No significant effect on p53 family members, *TP63* and *TP73.*

**Supplemental Figure 3 (Related to Figure 3)** (A) Workflow of the Seahorse XFe96 analyzer for OCR and ECAR readouts, 2-DG (2-deoxy-glucose). (B) Quantification of spare respiratory capacity in NES2shp53-1 and NES2shp53-2 NES cells compared to control cells. All data is presented as the mean ± SD of 4 independent experiments.

**Supplemental Figure 4 (Related to Figure 4)** (A) qRT-PCR validation of p53 KD during differentiation n=3 (B) Overview OXPHOS genes during differentiation, n=3. Data are presented as mean± SD, *p < 0,05. **p < 0,01.

**Supplemental Figure 5 (Related to Figure 5)** (A) Table of cell lines and shRNA constructs used in C9 and C10 iPS cells. (B) Western blot and quantification of p53 protein levels in C10 iPS cells before and after 3hr 0,5μM etoposide treatment, n=3 independent experiments. Values normalized to H3 levels and presented as Mean ± SD (C) Western blot analysis and quantification of phosphorylated p53-ser15 in C9 and C10 iPS cells in response to etoposide treatment. Values normalized to H3 levels and presented as Mean ± SD (D and E) Validation of iPS cell marker expression with immunofluorescence (D) and flow cytometry (E) of pluripotency markers OCT4, SSEA-4, SOX2, TRA 1-81 and TRA 1-60. (F) Unmerged NANOG images as shown in Fig. 5D. Scale bars 100μm.

**Supplemental Figure 6 (Related to Figure 6)** (A) Representative images of 10shCtrl and 10shp53-2 organoids where in C10shCtrl clear organized SOX2+ (green) neural tubular regions are visible surrounded by TBR1+ (red) TUJ1+ (red) neurons, whereas in C10shp53-2 TBR1 is almost absent. Scale bar 100μm. (B) Representative images of TBR1 (Magenta) and TBR2 (Yellow) staining in 30-day brain organoids. Scale bar 100μm. (C) Overview table of separate brain organoid inductions where disorganization was assessed as TBR1 present and well organized, or TBR1 absent and/or unorganized, and presented according to separate inductions. (D and E) representative images of cleaved Caspase 3 (red) and KI67 (red) immunofluourescence staining in D30 brain organoids, scale bar 100μm . (F) Representative EdU/PI flow cytometry plots showing the distribution of cells in G1, S and G2/M phase in C10 shCtrl and shp53 Day30 organoids pulsed with EdU for 90min (G) qRT-PCR validation of *TP53*KD in day 30 isolated PSA-NCAM+ progenitors, n=3 independent experiments. Data is presented as mean± SD, **p < 0,01. (H) OXPHOS genes qRT-PCR of C10 PSA-NCAM+ isolated neuronal progenitors, n=3 independent experiments. Data is presented as mean± SD, *p < 0,05.

**Supplemental video 1 (related to Figure 4E)** Video at 60 frames per second of NES1shCtrl after 15 days of differentiation, calcium is visualized with fluo-4 AM (see materials and methods).

**Supplemental video 2 (Related to Figure 4E)** Video at 60 frames per second of NES1shp53-2 after 15 days of differentiation, calcium is visualized with fluo-4 AM (see materials and methods).

**Supplemental Table 1. Oligonucleotide sequences used for qRT-PCR**

**Supplemental Table 2. Primary antibodies used and dilutions**

**Supplemental Table 3 (related to Figure 2)**

Transcriptome analysis of NES1 shCtrl and NES shp53-1 cells, in which we found 2159 genes deregulated (p<0,05, FC≥2) in NES1 shp53-1 cells compared to shCtrl.

**Supplemental Table 4 (related to Figure 2)**

List of deregulated pathways in NES1 shp53-1 cells compared to NES1 shCtrl cells.
