# Supplementary material for: p53 controls genomic stability and temporal differentiation of human neural stem cells and affects neural organization in human brain organoids
Source: Cell Death Dis. 2020 Jan 23;11(1):52. doi: 10.1038/s41419-019-2208-7 (PMC6978389; doi:10.1038/s41419-019-2208-7)
Supplement: Supplementary file 10 — Supplemental Table 1 [file 41419_2019_2208_MOESM10_ESM.docx]

**Supplemental Table 1. Oligonucleotide sequences used for qRT-PCR**

| Gene | Sequence 5’ -> 3’ |
| --- | --- |
| *TUBB3* Forward | CCTTTGGACATCTCTTCAGGC |
| *TUBB3* Reverse | CCACATCCAGGACCGAATCC |
| *DCX* Forward | GCAGTCTCCCATCTCTACGC |
| *DCX Reverse* | ATGGAATCACCAAGCGAGTC |
| *NDUFS5 Forward* | TGCACATGGAATCGGTTATACTC |
| *NDUFS5 Reverse* | CCGAAGCAAACACTCTACGAAAT |
| *DECR1 Forward* | CTGGACCCAACTGGAACATTT |
| *DECR1 Reverse* | GCAGCAAGATTTGCGAGTTCT |
| *ECH1 Forward* | ATAGTGGCTTCTCGCAGACTC |
| *ECH1 Reverse* | CAGTGAGGCGAAGGCTAATAC |
| *ACADVL Forward* | ACAGATCAGGTGTTCCCATACC |
| *ACADVL Reverse* | CTTGGCGGGATCGTTCACTT |
| *SDHD Forward* | TTGCTCTGCGATGGACTATTCC |
| *SDHD Reverse* | CAAGGCATCCCCATGAACAT |
| *HADHA Forward* | AAATTGACAGCGTATGCCATGA |
| *HADHA Reverse* | GCTTTCGCACTTTTTCTTCCACT |
| *COX7B Forward* | CTTGGTCAAAAGCGCACTAAATC |
| *COX7B Reverse* | AAAATCAGGTGTACGTTTCTGGT |
| *HK2 Forward* | GCTCTGGATCTTGGAGGGA |
| *HK2 Reverse* | CCAGGCATTCGGCAATGTG |
| *p53 Forward* | CCCCTCTGAGTCAGGAAACA |
| *p53 Reverse* | TCATCTGGACCTGGGTCTTC |
| *28s Forward* | TTGAAAATCCGGGGGAG |
| *28s Reverse* | ACATTGTTCCAACATGCC |
| *β2M Forward* | TGCTGTCTCCATGTTTGATGTATC |
| *β2M Reverse* | TCTCTGCTCCCCACCTCTAAGT |
