# Supplementary material for: p53 controls genomic stability and temporal differentiation of human neural stem cells and affects neural organization in human brain organoids
Source: Cell Death Dis. 2020 Jan 23;11(1):52. doi: 10.1038/s41419-019-2208-7 (PMC6978389; doi:10.1038/s41419-019-2208-7)
Supplement: Supplementary file 11 — Supplemental Table 2 [file 41419_2019_2208_MOESM11_ESM.docx]

**Supplemental Table 2. Primary antibodies used and dilutions**

| Target | Host | Company | Cat.Number | Dilution |
| --- | --- | --- | --- | --- |
| NANOG | Rabbit | Cell Signaling | 4903S | 1:200 ICC |
| NANOG | Rabbit | AbCam | [EPR2027(2)] (ab109250) | 1:1000 WB |
| p53 | Mouse | SantaCruz | sc-126 | 1:250/1:1000 IHC/WB |
| p53-ser15 | Rabbit | Cell Signaling | 9284S | 1:1000 WB |
| SSEA-4 | Mouse conjugated | BD Bioscience | 565977 | 1:20 FACS |
| OCT4 | Rabbit | Cell Signaling | C30A3 | 1:200 ICC |
| SOX2 | Rabbit | Millipore | AB5603 | 1:200 ICC |
| TRA1-81 | Mouse | AbCam | ab16289 | 1:500 ICC |
| TRA1-60 | Mouse conjugated-PE | BD Bioscience | 560884 | 1:15 FACS |
| SOX2 | Goat | SantaCruz | sc-17320 | 1:200 IHC |
| TBR1 | Rabbit | AbCam | AB31940 | 1:400 IHC |
| TUJ1 | Rabbit | Nordic biosite | 802001 | 1:500 ICC |
| TUJ1 | Mouse | Nordic biosite | 801202 | 1:500 IHC |
| EOMES | Mouse | Thermofisher | WD1928 | 1:400 IHC |
| KI67 | Rabbit | AbCam | Ab16667 | 1:500 IHC/ICC |
| H3 | Rabbit | AbCam | Ab1791 | 1:5000 WB |
| β-actin | Rabbit | AbCam | Ab8227 | 1:1000 WB |
| α -Tub | Mouse | SantaCruz | Gift from MAH | 1:10.000 WB |
| NESTIN | Mouse | Millipore | MAB 5326 | 1:500 ICC |
| PLZF | Mouse | ThermoFisher | MA5-15667 | 1:200 ICC |
| ZO-1 | Rabbit | ThermoFisher | 61-7300 | 1:500 ICC |
| γTub | Rabbit | Acris GmbH | APO06470PU-N | 1:250 ICC |
| TRA-1-60-PE | Mouse | BD Biosciences | 560884 | 1:20 FACS |
| V450 SSEA-4, Clone MC813-70 | Mouse | BD Biosciences | MC813***-***70 | 1:20 FACS |
| DECR1 | Rabbit | AbCam | ab95965 | 1:1000 WB |
| PSA-NCAM-microbeads | Mouse | Miltenyi Biotec | 130-092-966 | 20ul per 10^7^ cells MACS |
